# *In vitro* and intracellular inhibitory activities of nosiheptide against *Mycobacterium abscessus*

**DOI:** 10.3389/fmicb.2022.926361

**Published:** 2022-07-26

**Authors:** Rui Zhu, Xia Yu, Tingting Zhang, Yaoyao Kong, Fen Wang, Junnan Jia, Yi Xue, Hairong Huang

**Affiliations:** National Clinical Laboratory on Tuberculosis, Beijing Key Laboratory on Drug-Resistant Tuberculosis, Beijing Chest Hospital, Capital Medical University, Beijing, China

**Keywords:** *Mycobacterium abscessus*, nosiheptide, intracellular bactericidal activity, susceptibility, cytotoxicity

## Abstract

The high level of inherent drug resistance of *Mycobacterium abscessus* makes the infection caused by it very difficult to be treated. The objective of this study was to evaluate the potential of nosiheptide (NOS) as a new drug candidate for treating *M. abscessus* infections. The microplate AlamarBlue assay was performed to determine the minimum inhibitory concentrations (MICs) of NOS for 28 reference strains of rapidly growing mycobacteria (RGM) and 77 clinical isolates of *M. abscessus*. Time-kill kinetic and post-antibiotic effect (PAE) of NOS against *M. abscessus* was evaluated. Its bactericidal activity against *M. abscessus* in macrophages was determined by an intracellular colony numerating assay. NOS manifested good activity against the reference strains of RGM and *M. abscessus* clinical isolates *in vitro*. The MICs of NOS against *M. abscessus* clinical isolates ranged from 0.0078 to 1 μg/ml, and the MIC_50_ and MIC_90_ were 0.125 μg/ml and 0.25 μg/ml, respectively. The pattern of growth and kill by NOS against *M. abscessus* was moderate with apparent concentration-dependent characteristics, and the PAE value of NOS was found to be ~6 h. Furthermore, NOS had low cell toxicity against the THP-1 cell line after 48 h of exposure (IC_50_ = 106.9 μM). At 4 μg/ml, NOS exhibited high intracellular bactericidal activity against *M. abscessus* reference strains with an inhibitory rate of 66.52% ± 1.51%, comparable with that of clarithromycin at 2 μg/ml. NOS showed suitable inhibitory activities against *M. abscessus in vitro* and in macrophages and could be a potential drug candidate to treat *M. abscessus* infection.

## Introduction

Nontuberculous mycobacteria (NTM) are infecting populations at increasing rates in China and globally (Brode et al., 2017; Baldwin et al., 2019). Many species of NTM have been reported and are categorized into rapidly growing mycobacteria (RGM) and slowly growing mycobacteria (SGM) according to their speed of growth (i.e., the appearance of visible colonies on a solid medium within or after 1 week). *Mycobacterium abscessus* complex is the most frequently isolated RGM in the clinical specimen in China, and it mainly causes pulmonary disease, while soft tissue infections are also encountered frequently (Compain et al., 2018). *M. abscessus* is notorious for its high level of inherent antibiotic resistance; therefore, its infections are usually difficult to be treated (Ferro et al., 2015; Le Run et al., 2019). The sustained sputum culture conversion rate of pulmonary disease caused by *M. abscessus*, when treated with clarithromycin (CLA) containing regimen, was 34% while it was only 20% for the refractory cases (Pasipanodya et al., 2017). Therefore, *M. abscessus* pneumonia is named “antibiotic nightmare” (Nessar et al., 2012). The limited availability and efficacies of the few available medication choices highlight the desperate need for new or repurposed drugs to change this clinical predicament.

Nosiheptide (NOS), a sulfur-containing polypeptide antibiotic produced by *Streptomyces actuosus*, has been widely used as an antimicrobial feed additive in animal food production to improve feed efficiency and promote feed efficiency in poultry and pigs without leaving any residues in the body (Benazet and Cartier, 1980; Cromwell et al., 1984). A previous study showed that NOS possessed substantial activities against Gram-positive bacteria, including methicillin-resistant *Staphylococcus aureus*, penicillin-resistant *Streptococcus pneumonia*, and vancomycin-resistant *enterococci* (Haste et al., 2012). Furthermore, NOS also demonstrated potent inhibitory activity *in vitro* against some SGM species, including *Mycobacterium avium, Mycobacterium intracellulare*, and *Mycobacterium bovis* (Hosoda et al., 2019).

To better understand the antimicrobial activity of NOS against RGM, we determined the minimum inhibitory concentrations (MICs) of 28 RGM reference strains and 77 clinical isolates of *M. abscessus* collected in Beijing, China. Furthermore, *M. abscessus* time-kill kinetic and the post-antibiotic effect (PAE) of NOS were characterized, while the bactericidal activity against *M. abscessus* in macrophages was also analyzed. Our results indicated that the rediscovery of the current antibiotics can supply a meaningful strategy to screen therapeutic candidates for *M. abscessus*, which is considered one of the pathogens with the most severe drug resistance.

## Materials and methods

### Ethics statement

As the study only involved laboratory testing of mycobacterial strains without the direct involvement of human subjects, ethical approval was not sought.

### Bacterial strains and culture conditions

The mycobacterial reference strains used in this study were stored in the Bio-bank in Beijing Chest Hospital. These reference strains were obtained either from the American Type Culture Collection (ATCC) or the German Collection of Microorganisms (DSM). The species constitution of the tested strains is listed in Table 1.

**Table 1:** Minimum inhibitory concentrations (MICs) of nosiheptide (NOS) against reference strains of 28 rapidly growing mycobacterium (RGM) species.

**Strain by Mycobacterium type**	**Species (strain)**	**MIC (**μ**g/ml) by antimicrobial agent**
		NOS
ATCC 19977	*Mycobacterium abscessus*	0.125
ATCC 27406	*Mycobacterium agri*	<0.008
ATCC 27280	*Mycobacterium aichiense*	>8
ATCC 23366	*Mycobacterium aurum*	4
ATCC 33464	*Mycobacterium austroafricanum*	4
ATCC 14472	*Mycobacterium chelonae*	2
ATCC 19627	*Mycobacterium chitae*	0.125
ATCC 27278	*Mycobacterium chubuense*	2
DSM 44829	*Mycobacterium cosmeticum*	>8
ATCC 19340	*Mycobacterium diernhoferi*	0.063
ATCC 35219	*Mycobacterium fallax*	1
ATCC 14474	*Mycobacterium flavescens*	0.5
ATCC 6841	*Mycobacterium fortuitum*	>8
ATCC 27726	*Mycobacterium gadium*	0.031
ATCC 43909	*Mycobacterium gilvum*	<0.008
ATCC BAA-95	*Mycobacterium goodii*	>8
ATCC 25795	*Mycobacterium neoaurum*	0.125
ATCC 27023	*Mycobacterium obuense*	4
ATCC 19686	*Mycobacterium parafortuitum*	2
DSM 43271	*Mycobacterium peregrinum*	>8
ATCC 11758	*Mycobacterium phlei*	>8
ATCC 35154	*Mycobacterium pulveris*	0.125
ATCC 35796	*Mycobacterium senegalense*	2
ATCC 33027	*Mycobacterium sphagni*	0.063
ATCC 19420	*Mycobacterium smegmatis*	4
ATCC 19527	*Mycobacterium thermoresistibile*	0.5
ATCC 27282	*Mycobacterium tokaiense*	>8
ATCC 15483	*Mycobacterium vaccae*	0.25

The *M. abscessus* clinical strains, isolated from the patients with pulmonary infections, were classified as NTM preliminarily with a p-nitrobenzoic acid-containing medium and then were identified at the species level by sequence alignment of *16S rRNA, hsp65, rpoB*, and *16-23S rRNA* internal transcribed spacer sequence in parallel (Lee and Whang, 2017). All the strains were stored at −80°C freezer and were subcultured on solid Löwenstein-Jensen medium at 37°C.

### MIC testing

Nosiheptide and CLA were purchased from Toronto research chemicals (Canada, CAS: 56377-79-8, Cat No.: N884000) and Solarbio (China), respectively. The above antibiotics were dissolved in dimethyl sulfoxide (DMSO, Sigma, USA) at an 8 mg/ml concentration and stored at −20°C. The broth microdilution method was performed according to the guidelines from the Clinical and Laboratory Standards Institute (CLSI) (Wayne, 2011). Cation-adjusted Mueller-Hinton broth (CAMHB) was used for the MIC test of RGM. Final concentrations of NOS in titration reaction ranged from 0.0078 to 8 μg/ml by serially 2-fold diluted in a 96-well microtiter plate. Cultures were scraped from LJ media, homogenized, then adjusted to McFarland 0.5 with sterile saline, and diluted 200-fold for inoculation. A volume of 100 μl of diluted inoculum was added to each well. The plates were incubated at 37°C for 3 days. Notably, 20 μl of AlamarBlue (BD, USA) and 50 μl of 5% Tween 80 (Sangon Biotech, China) were added to each well, and the plates were re-incubated at 37°C for an additional 24 h before assessing color change. The blue color indicated no cell growth, and the pink color indicated mycobacterial growth (Coeck et al., 2016). The MIC was defined as the lowest concentration of drug that inhibited color change from blue to pink. CLA was used as a positive control, and the reported MICs of *M. abscessus* ATCC19977 to CLA are 0.0625 or 0.125 μg/ml in different batches.

### Minimum bactericidal concentration against *M. abscessus*

Minimum bactericidal concentrations (MBCs) of NOS and CLA were determined to distinguish bacteriostatic activity from bactericidal activity. *M. abscessus* reference strains (ATCC 19977) and five clinical isolates of *M. abscessus*, including two with smooth morphology and three with rough morphology, were tested. After 4 days of incubation with drugs at different concentrations, the wells in which the drug concentrations were higher than the MIC were tested, and 100 μl medium from each well was serially diluted (by 10-fold) and inoculated onto Mueller Hinton (MH) agar plate. The colony-forming units (CFUs) were counted after 5 days of incubation at 37°C. The MBC value was defined as the drug dilution, which resulted in a 99.9% decreased CFU in contrast to the initial bacterial load (Ling et al., 2015). An antibiotic was considered bactericidal when the MBC/MIC ratio was ≤ 4; otherwise, it was considered bacteriostatic (Zhang et al., 2020).

### FACS analysis by flow cytometry

Cell membrane integrity was determined by the propidium iodide (PI)-based method as previous report (Baindara et al., 2016). *M. abscessus* (ATCC 19977) cells were grown to the mid-exponential growth phase and centrifuged at 8,000 rpm for 10 min, and the cell pellet was washed three times with phosphate-buffered saline (PBS). The pellet was diluted with PBS to a cell density of 10^6^ CFU/ml. Subsequently, it was treated with 4 μg/ml of NOS or CLA separately and incubated at 37°C for 1, 3, and 6 h. Cells treated with PBS were used as untreated control. The cell pellets were thoroughly washed three times with PBS and treated with PI (20 μg/ml) for 15 min. Cells were centrifuged, and the pellet was washed with PBS three times to remove excess PI. Cells were fixed with 4% paraformaldehyde for 20 min at room temperature. Subsequently, the samples were immediately analyzed using a flow cytometer (BD LSRFortessa, USA).

### Time-kill assay

The reference strain of *M. abscessus* was prepared fresh for each experiment by growing over 24 h in CAMHB containing 0.05% Tween 80, to obtain bacteria in the early logarithmic phase of growth. Tubes (5 ml) of CAMHB, containing NOS or CLA at 1 × MIC, 2 × MIC, 5 × MIC, 10 × MIC, or 20 × MIC, were inoculated with 5 ×10^5^ CFU/ml of bacterial cells. The tubes were incubated with shaking at 200 rpm/min for 5 days at 37 °C. A growth control tube, containing only bacteria (no antibiotics), as well as a sterility control (medium only), was included. The bacteria were enumerated at defined time intervals (12, 24, 48, 72, 96, and 120 h) by plating serial dilutions on MH agar plates. CFUs were enumerated after an additional 5 days of incubation at 37°C. A CFU count curve was drawn over time to characterize the effect of the antibiotics in different treatment concentrations.

### Post-Antibiotic effect

The PAE was determined as described previously (Islam et al., 2019). In Middlebrook 7H9 broth with 0.05% Tween 80, *M. abscessus* (ATCC 19977) in the early log phase [optical density at 600 nm (OD_600_) = 0.2] was exposed to NOS or CLA at the same concentration of 10 μg/ml. The same density of bacterial cells with DMSO but no drug was used as a positive control, whereas fresh medium without any bacteria and drug was used as a negative control. The cultures were incubated at 37°C under shaking conditions for 2 h. Antibiotics were removed by centrifugation and washing three times with pre-warmed Middlebrook 7H9 broth supplemented with 0.05% Tween 80. Then, the cultures were incubated at 37 °C with shaking until they reached maximum OD_600_ (OD max). The OD_600_ of each culture was determined every 24 h after drug removal. PAE was defined as the time taken for the antibiotic-treated culture to reach 50% OD_max_ of the drug-free culture minus the time taken for the drug-free control to reach the same point (Islam et al., 2019).

### Cytotoxicity assay

THP-1 was purchased from the Wuhan Procell Life Science & Technology Co., Ltd and used to evaluate the cytotoxicity of NOS. Cells were seeded into 96-well plates at a density of 3 ×10^4^ cells/well and differentiated into macrophages with phorbol myristate acetate (PMA) at a final concentration of 200 ng/ml at 37°C with 5% CO_2_ (Leinardi et al., 2020). After 48 h, cells were washed twice with PBS. Different concentrations (2.5 μM, 5 μM, 10 μM, and 20 μM) of NOS, diluted in RPMI-1640 medium with 10% fetal bovine serum (FBS), were added into the wells and incubated for an additional 24 h and 48 h. DMSO was used as a negative control. NSC 228155 (MedChemExpress, USA), as an activator of EGFR and was reported to be toxic in breast cancer MDA MB468 cell line, was used as a positive control at a final concentration of 2.5 μM (Zhao et al., 2020). The cytotoxicity of THP-1 was measured following the instructions in the manual provided in the cell counting kit-8 (CCK-8, Solarbio, China). After carefully removing the cell culture medium, 10 μl of CCK8 and 90 μl of RPMI-1640 medium (without 10% FBS) were added to each well and kept at 37°C for 2 h. Then, the absorbance was measured at 450 nm wavelength using the Multiskan FC microplate reader (Thermo Fisher, USA). The cell survival rate was calculated by the following equation: cell survival rate (%) = (absorbance of the experimental group-background absorbance)/(absorbance of the control group-background absorbance) ×100%. In addition, the cytotoxicity was also determined using undifferentiated THP-1 cells.

### Intracellular killing and concentration-kill assay

The THP-1 cells were seeded at 5 ×10^5^ cells/well in a 24-well plate and induced to differentiate into macrophages with PMA (200 ng/ml) for 48 h. The cells were infected at a multiplicity of infection (MOI) of 1:1 or 10:1 with *M. abscessus* (ATCC 19977) suspended in RPMI 1640 medium with 10% FBS. After 4 h of infection at 37 °C in 5% CO_2_, the cells were gently washed three times with pre-warmed 1 × PBS to remove the extracellular bacteria. Amikacin (200 μg/ml) diluted in RPMI 1640 medium with 10% FBS was added to kill the remaining extracellular bacteria for an additional 2 h (Chen et al., 2021). NOS was added to the corresponding well at 4, 2, and 1 μg/ml, and CLA at the same respective concentrations was used as a positive control, while a culture medium with DMSO was used as a negative control. The synergistic bactericidal effect was also evaluated using a combination of NOS (1 μg/ml) and CLA (1 μg/ml). After 48 h, the cells were lysed with 0.01% Triton X-100 and then serially diluted with 1 × PBS. The number of CFUs was quantified by plating them onto MH agar plates. The percentage of intracellular bacterial survival rate was calculated by the following equation: viability = CFU of bacteria under the treatment of NOS or CLA/CFU of bacteria treated with DMSO ×100%.

### Statistical analysis

All the experiments were performed in triplicate. The tentative epidemiological cutoff (ECOFF) was determined according to the distribution profile of the MIC values. For the unimodal MIC distribution profile, ECOFF was defined as the concentration that could inhibit 95% of the bacterial population; for the bimodal MIC distribution profile, ECOFF was set between the two populations (Yu et al., 2019). Statistical analyses were performed using GraphPad Prism 9.0 (GraphPad Software Inc., La Jolla, CA, USA). For cytotoxicity and intracellular bactericidal assay, one-way ANOVA was used to determine significant differences between the groups. The calculation of the half-maximal inhibitory concentration (IC_50_) values of NOS on the THP-1 cell line was performed by nonlinear regression.

## Results

### MICs of NOS against the reference strains

The MICs of the 28 RGM reference strains against NOS are presented in Table 1. NOS exhibited good *in vitro* activity against the recruited RGM reference strains. Notably, 17 out of 28 RGM species had MICs ≤ 2 μg/ml, and 12 of them had MICs ≤ 0.5 g/ml. Among the 11 remaining RGM species, 7 had MICs of >8 μg/ml, whereas the other four had MICs of 4 μg/ml.

### MBCs of NOS against *M. abscessus* isolates

**Figure 1:**
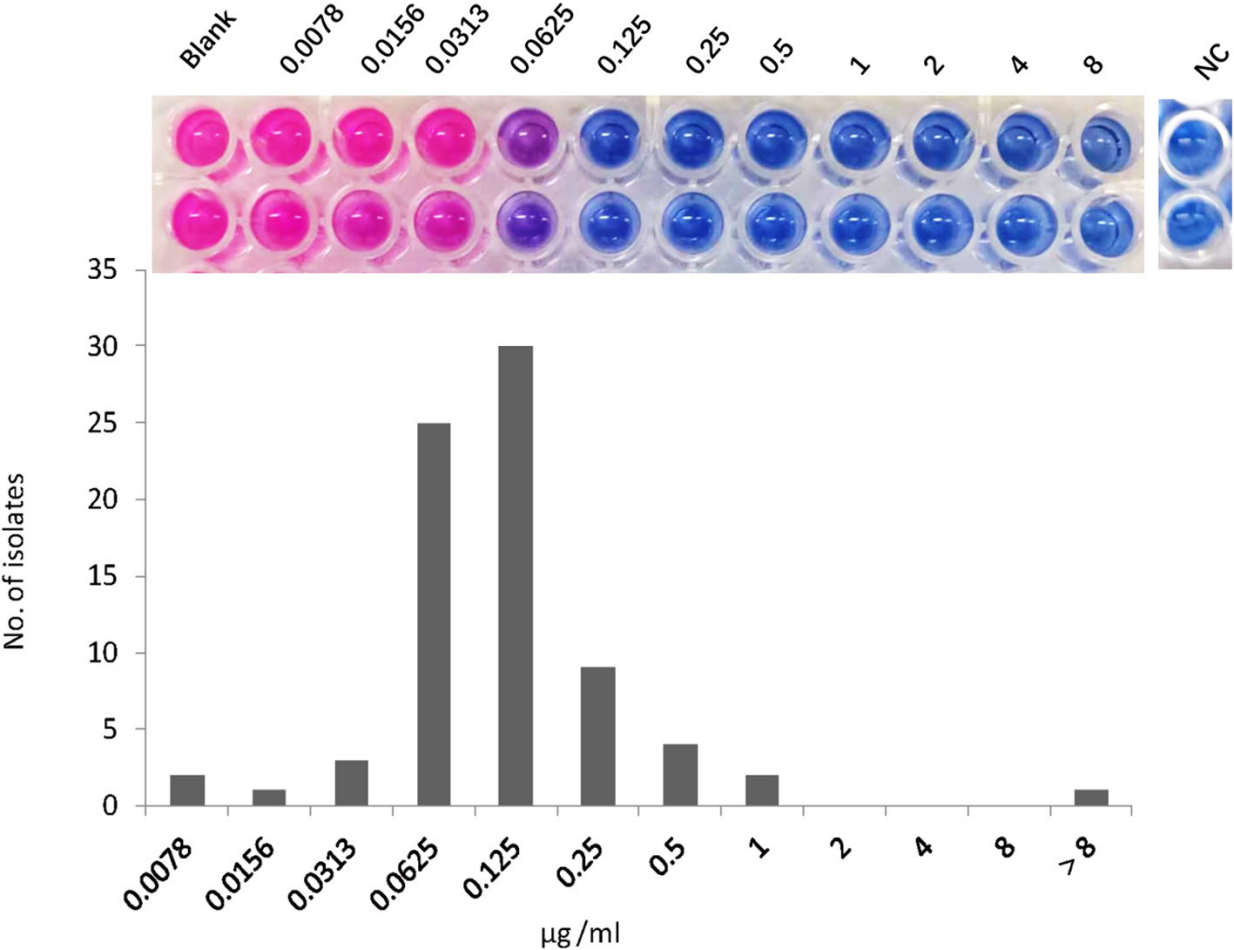
The minimum inhibitory concentration (MIC) distribution of nosiheptide (NOS) against *Mycobacterium abscessus* isolates. The MIC was determined by microtiter plate AlamarBlue assay. The MIC outcome of NOS against *M. abscessus* ATCC19977 was on the top. The blank group: only bacteria without drugs; NC group: negative control group, this group was free of bacteria and drugs to prove that the experiment was not contaminated.

The MICs of NOS against the reference strain of *M. abscessus* were 0.125 μg/ml. The MIC distribution of the 77 *M. abscessus* clinical isolates is shown in Figure 1. Except for one isolate with MIC >8 μg/ml, the MICs of NOS against *M. abscessus* clinical isolates ranged from 0.0078 μg/ml to 1 μg/ml. The MIC_50_ and MIC_90_ were 0.125 μg/ml and 0.25 μg/ml, respectively. NOS exhibited potent activity against *M. abscessus* with a tentative ECOFF of 0.5 μg/ml, whereas the overwhelming majority of the isolates (79.2%, 61/77) had MICs ≤ 0.125 μg/ml.

The MBC/MIC ratios of NOS against *M. abscessus* reference strains and 5 clinical isolates ranged from 32 to 128, while these of CLA ranged from 8 to 32 (Table 2). Since both NOS and CLA had MBC/MIC ratios >4 against the recruited strains, the bacteriostatic effect was then recognized for them.

**Table 2:** Minimum inhibitory concentration (MIC), minimum bactericidal concentration (MBC) values, and antibacterial activities of NOS and clarithromycin (CLA) against *Mycobacterium abscessus*.

**Isolate**	**Morphotype**			**NOS (**μ**g/ml)**			**CLA (**μ**g/ml)**
		MIC	MBC	MBC/MIC ratio	MIC	MBC	MBC/MIC ratio
ATCC19977	Smooth	0.125	8	64	0.125	2	16
121	Smooth	0.0625	4	64	0.25	8	32
119	Smooth	0.03125	4	128	0.03125	0.5	16
106	Rough	0.125	8	64	0.0625	1	16
108	Rough	0.0625	2	32	0.0625	0.5	8
120	Rough	0.0625	4	64	0.03125	0.5	16

### FACS analysis by flow cytometry

**Figure 2:**
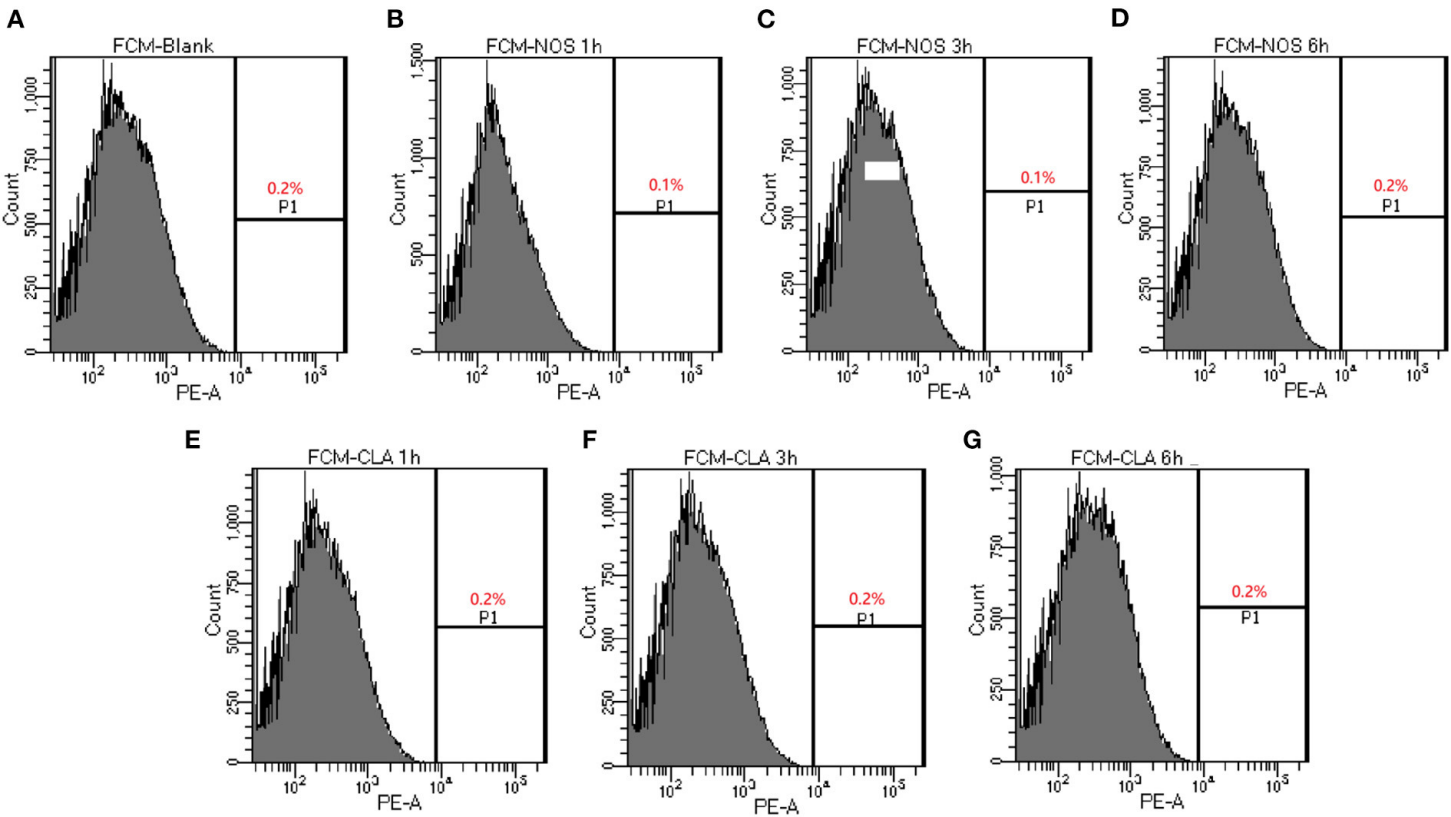
Determination of membrane-permeabilizing properties of NOS by FCM of *M. abscessus* ATCC 19977. Bacterial cells are treated with NOS and clarithromycin (CLA) in a time-dependent manner. The percentage of bacterial cells shows the increased uptake of propidium iodide with increasing time intervals. **(A)** Bacterial cells without treatment serve as a negative control. **(B–D)** The results after 1, 3, and 6 h of treatment with NOS (4 μg/ml). **(E–G)** The results after 1, 3, and 6 h of treatment with CLA (4 μg/ml). Samples were prepared in phosphate-buffered saline (PBS).

The membrane integrity experiment was determined using nucleic acid stain PI. Compared with a negative control with PBS, up to 0.2 % of cells showed PI uptake in NOS (4 μg/ml, 32 × MIC) or CLA (4 μg/ml, 64 × MIC) groups, respectively, which hints the integrity of the cell membrane that was not destroyed after treatment up to 6 h (Figure 2). These data suggested that both NOS and CLA worked as a bacteriostatic agent for *M. abscessus*

### Time-kill assay

**Figure 3:**
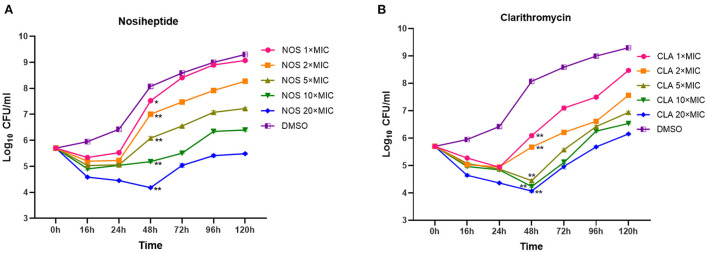
Time-kill kinetics of **(A)** NOS and **(B)** CLA against *M. abscessus* ATCC 19977. Antibiotic concentrations are presented as different symbols. Dimethyl sulfoxide (DMSO) was used as a negative control, and CLA was used as a positive control. *P < 0.05; **P < 0.01.

In general, the patterns of growth and killing by NOS and CLA against *M. abscessus* were moderate with apparent concentration-dependent features (Figure 3). Killing effects were observed at all the tested concentrations in the first 16 h drug for both NOS and CLA. Within 16 h−48 h explosion, only the exposure concentrations at 20 × MIC of NOS could effectively decrease the bacterial density. In contrast, concentrations at 20 × MIC, 10 × MIC, and 5 × MIC of CLA presented killing effects within 16 h−48 h drug explosion. However, regrowth was observed with all the tested concentrations after 48 h exposure for both NOS and CLA. On day 5, NOS acquired a mean 0.22 log_10_ CFU ml^−1^ decrease, whereas CLA had a 0.45 log_10_ CFU ml^−1^ increase at 20 × MIC of each antibiotic, compared with the initial inoculum (5.48 log_10_ CFU ml^−1^).

### The PAE of NOS and CLA against *M. abscessus*

**Figure 4:**
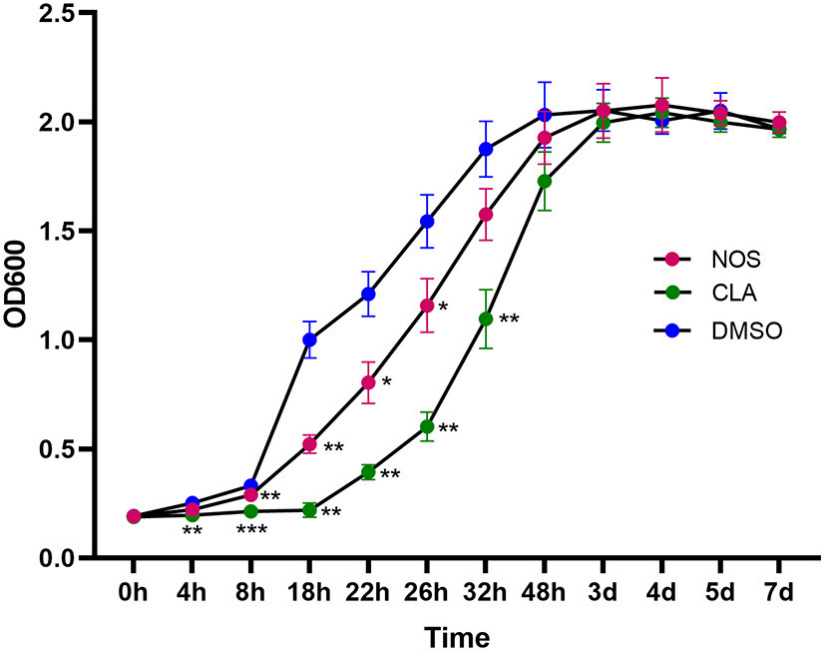
Post-antibiotic effect (PAE) in the growth of *M. abscessus* ATCC19977 after pulse dosing with NOS and CLA. CLA was used as a positive control and DMSO as a negative control. All data are shown as the means ± SD (*n* = 3). NOS vs. DMSO. *P < 0.05; **P < 0.01; ***P < 0.001.

Following 2 h of exposure to 10 μg/ml of NOS or CLA, the retarded growth of *M. abscessus* was monitored. The PAE value of NOS was found to be approximately 6 h, which was shorter than CLA (12 h) (Figure 4).

### Cytotoxicity assay

**Figure 5:**
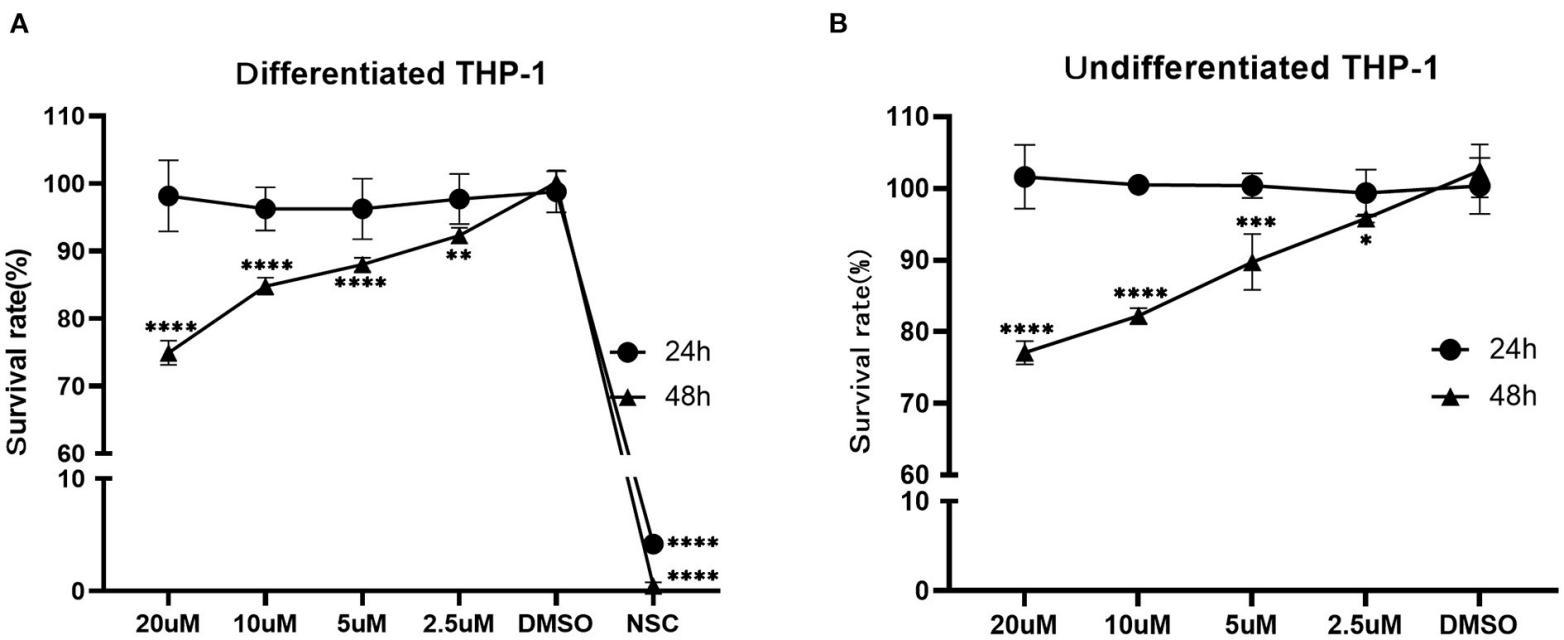
Cytotoxicity assay of NOS in the differentiated **(A)** and undifferentiated **(B)** THP-1 cell lines. DMSO group: the negative control; NSC 228155 group: the positive control (at a final concentration of 2.5 μM). All data are shown as the means ± SD (*n* = 3). *P < 0.05; **P < 0.01; ***P < 0.001; ****P < 0.0001.

According to the CCK8 assay, the survival rate of the differentiated THP-1 when exposed to NSC 228155 (2.5 μM), which was used as a positive control, was 4.19% (24 h) and 0.47 % (48 h), respectively. The IC_50_ of NOS against the differentiated and undifferentiated THP-1 cell lines for 48 h of exposure were ~106.9 and 91.8 μM, respectively. When the concentration of NOS was lower than 20 μM, the survival rates of the differentiated and undifferentiated THP-1 were nearly 100% within 24 h of the exposure. When extending the incubation time to 48 h, the survival rate of the differentiated THP-1 was comparable with that of DMSO with a NOS concentration lower than 2.5 μM (i.e., 3.06 μg/ml, equal to 24 times MIC of *M. abscessus* reference strain ATCC 19977). With the NOS concentration at 10 μM or 20 μM for 48 h exposure, the differentiated THP-1 cells' survival rates were 84.80 and 74.94%, respectively. Additionally, the undifferentiated THP-1 cells' survival rates were 82.20 and 77.07%, respectively (Figure 5).

### Intracellular killing and concentration-kill assay

**Figure 6:**
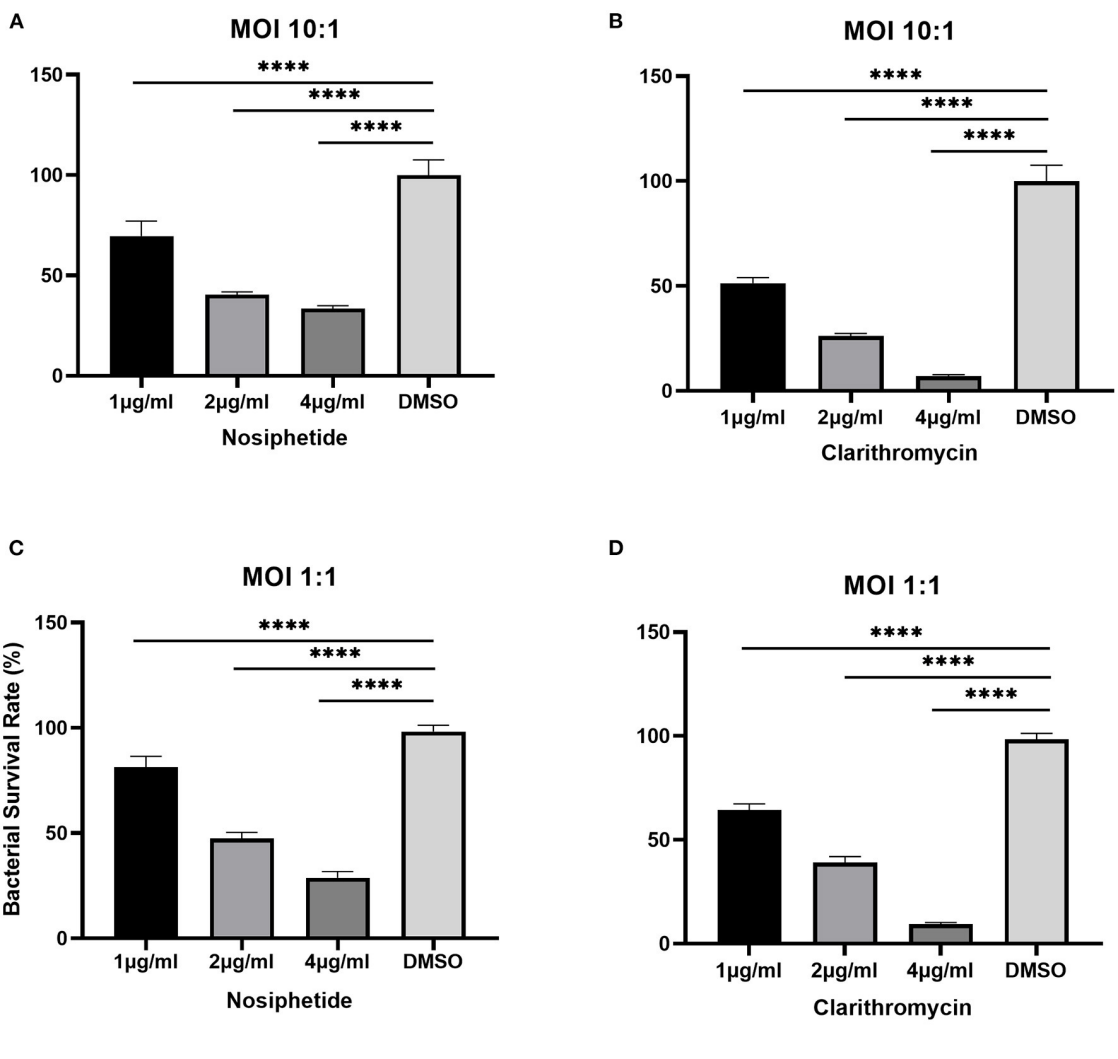
Intracellular bactericidal activities of different concentrations of nosiheptide (NOS) and CLA (the positive control) against *M. abscessus* ATCC 19977 in macrophages. **(A)** NOS at an MOI of 10:1. **(B)** clarithromycin at an MOI of 10:1. **(C)** NOS at an MOI of 1:1. **(D)** clarithromycin at an MOI of 1:1. DMSO group: the negative control. All data are shown as the means ± SD (*n* = 3). ****P < 0.0001.

**Figure 7:**
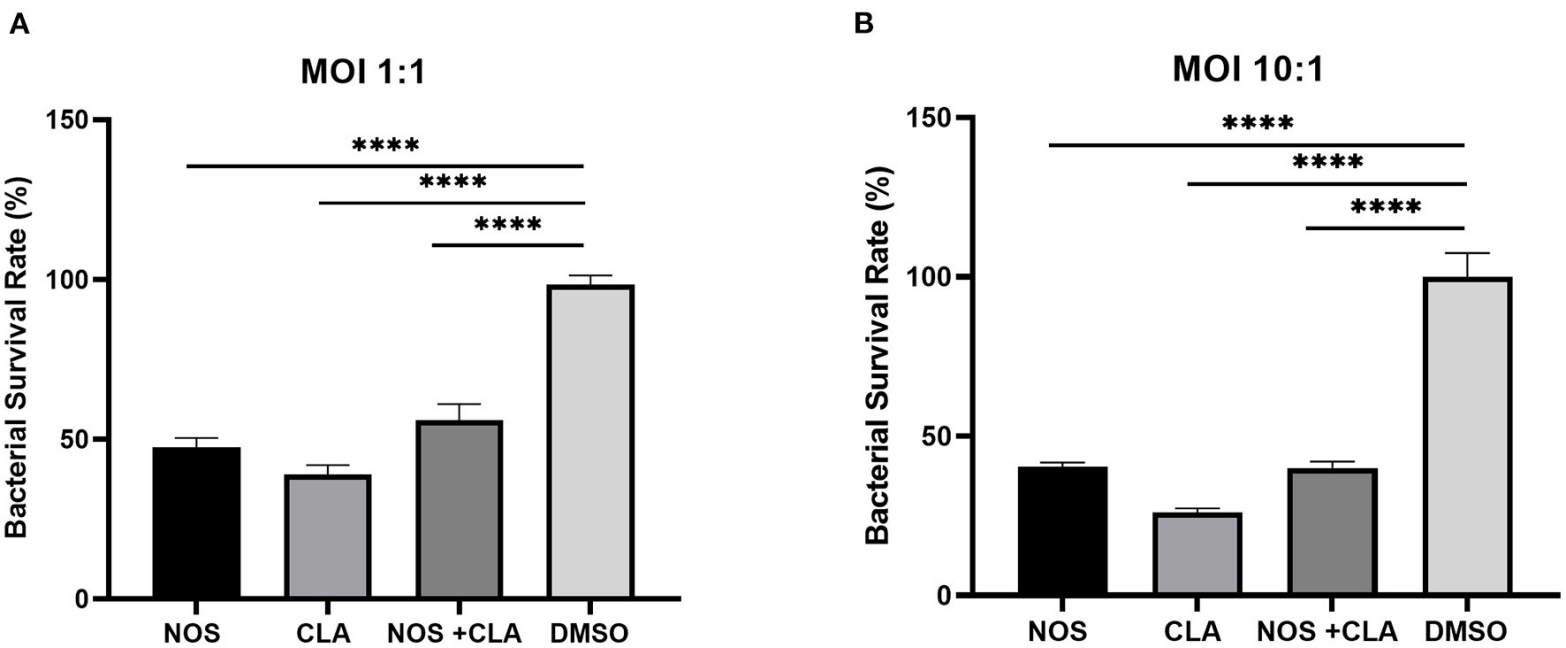
The intracellular bacterial survival rate of *M. abscessus* ATCC 19977 with NOS exposure. **(A)** At an MOI of 1:1. **(B)** at an MOI of 10:1. NOS group: infected macrophage treated with NOS (2 μg/ml); CLA group: the positive control, infected macrophage treated with CLA (2 μg/ml); NOS + CLA group: infected macrophage treated with NOS (1 μg/ml) and CLA (1 μg/ml); DMSO group: the negative control. All data are shown as the means ± SD (*n* = 3). ****P < 0.0001.

The bacterial survival rate was quantitated as shown in Figure 6. Either at MOI = 1:1 or MOI = 10:1, obvious reductions in CFU number were observed when treated with NOS compared with the DMSO treatment. At MOI of 10:1, NOS at 4 μg/ml inhibited 66.52 ± 1.51% of intracellular bacterial growth, which was comparable with CLA at 2 μg/ml (i.e., 73.91 ± 1.31%). A similar outcome was acquired at MOI of 1:1, and the inhibitory rate of NOS at 4 μg/ml and CLA at 2 μg/ml was 71.19 ± 2.94% and 61.02 ± 2.93%, respectively. Furthermore, the NOS + CLA group (1 μg/ml NOS and 1 μg/ml CLA) inhibited 60.00 ± 2.00% bacterial growth at MOI of 10:1. Additionally, the inhibitory rates of NOS (2 μg/ml) and CLA (2 μg/ml) at MOI of 10:1 were 59.57 ± 1.31 and 73.91 ± 1.31%, respectively (Figure 7). Thus, no obvious synergistic effect was detected between NOS and CLA against *M. abscessu*s.

## Discussion

A previous study showed that NOS was effective against *M. avium* complex with MIC ranging from 0.012 to 0.024 μg/ml (Hosoda et al., 2019), which increases interest in its potential inhibitory activity against other Mycobacteria. Our study focused on the evaluation of the antimicrobial activity against *M. abscessus*, which is one of the most frequently isolated RGM species with extremely difficult treatment. In this study, NOS demonstrated antimicrobial activities against many of the recruited reference strains of RGM, and 12 out of 28 strains had MICs ≤ 0.5 g/ml. Strikingly, NOS manifested more potent activities against the clinical isolates of *M. abscessus*. The MIC_50_ and MIC_90_ of NOS against *M. abscessus* were 0.125 and 0.25 μg/ml, respectively. These low MICs make NOS very promising to be an antimicrobial candidate. Even though the MBC/MIC ratio of NOS for *M. abscessus* is suggestive of its bacteriostatic activity, a good prospect for it could still be expected, as CLA also presented to be a bacteriostatic reagent in this study.

Previous studies had demonstrated that linezolid and amikacin all lacked antimicrobial activity against *M. abscessus* by time-kill kinetics. None of the tested concentrations (up to 32 × the MIC) led to a significant reduction in CFU number during the first 24 h of exposure (Maurer et al., 2014; Ferro et al., 2015). Consistent with these results, poor treatment outcomes with regiments containing the above drugs for *M. abscessus* infection were obtained (van Ingen et al., 2013; Baker et al., 2021). Intriguingly, NOS showed a moderate killing effect against *M. abscessus* within the first 16 h at a concentration equal to or higher than 1 × MIC. However, except at the concentration of 20 × MIC, the tested concentrations could not inhibit the growth after 16 h. This was in accordance with the data of PAE, which showed that NOS has a short PAE of approximately 6 h, which hints that this antibiotic need to be administrated with short dosing intervals. Surprisingly, as a cornerstone of *M. abscessus* therapy, CLA did not show a high killing effect in our study either. Although the antibacterial activity of CLA was superior to NOS in the first 24 h, the bacterial load of 120 h in the CLA group was higher than that of NOS (i.e., a mean 0.45 log_10_ CFU ml^−1^ increase vs. 0.22 log_10_ CFU ml^−1^ decrease at 20 × MIC of antibiotics compared with the initial inoculum). Furthermore, NOS (4 μg/ml, 32 × MIC) inhibited 66.52 ± 1.51% of intracellular bacterial growth at MOI of 10:1 after 48 h incubation. Taken together, NOS demonstrated a moderate killing effect both in time-kill kinetic and intracellular bacterial growth tests, which may aid in designing a more active treatment regimen against *M. abscessus* infection.

Nosiheptide inhibits protein synthesis by binding to the L11-binding domain of 23S ribosomal RNA (Harms et al., 2008). A previous study showed that NOS was an inhibitor of protein translation, and notably, it had 100-fold greater efficacy on *Escherichia coli* (IC_50_ = 0.23 μM) than on eukaryotic cells of rabbit reticulocyte (IC_50_ > 200 μM) (Lentzen et al., 2003). This outcome suggested that this compound was less toxic to eukaryotic cells. In our study, cytotoxicity assays indicated that the differentiated/undifferentiated THP-1 cells had nearly 100% viability when exposed to NOS for 24 h below 20 μM (equal to 24.4 μg/ml), which was approximately 200-fold above the MIC of the reference strain of *M. abscessus*. Furthermore, it was reported that NOS was noncytotoxic when incubated for 72 h with the cervical carcinoma HeLa cell line at up to 128 μg/ml, which is approximately 1,000-fold above the MIC value of *M. abscessus* (Haste et al., 2012). These data obtained from our or other studies, plus the fact that NOS is used as an antimicrobial feed additive in animal husbandry, all favor the safety of using NOS as a medication.

Given the good inhibitory activity and the low cytotoxicity of NOS *in vitro*, the *in vivo* efficacy of this compound is worthy of investigation. Limited data exist on the therapeutic potential of this compound. A recent study demonstrated that the NOS exhibited therapeutic efficacy against *M. abscessus in vivo*-mimic silkworm infection assay with 50% effective dose (ED50)/MIC = 9.36 (Hosoda et al., 2020). The ED50/MIC ratio of a compound is an index of drug potential, and the ratio below 10 is typically indicative of clinical usefulness (Hamamoto et al., 2004). In addition, Haste et al. showed that NOS treatment (20 mg kg^−1^, i.p. at 1 and 8 h postinfection) demonstrated *in vivo* activity in a murine model of methicillin-resistant *Staphylococcus aureus* infection, i.e., provided significant protection against mortality, and only one out of ten mice in the NOS group died (Haste et al., 2012). All these data strongly supported that NOS was efficacious *in vivo* as well. A further study focusing on the pharmacokinetic/pharmacodynamic (PK/PD) profile of NOS is needed to gain more insights into its potential as a new antibiotic.

There are some limitations in our study. First, all the tested clinical isolates were collected from a single institution. It may not represent all the characteristics of the *M. abscessus* isolates. Second, the bactericidal activity of NOS against *M. abscessus* was only investigated *in vitro* and in macrophages, and these might not fully reflect the real reaction *in vivo*. Further studies are required to investigate its efficacy in an animal model as well as its PK/PD profile. Third, the clinical application of NOS is largely hindered due to its poor water solubility and low bioavailability (Fan et al., 2021). Thus, the development of novel applications, appropriate administration, or improved analogs for NOS would be necessary and effective to overcome the obstacles faced in clinical application.

In conclusion, both *in vitro* and intracellular bacterial growth testing demonstrated that NOS has a strong antimicrobial activity against *M. abscessus* with low cytotoxicity and, therefore, could be considered as a promising drug candidate for *M. abscessus* treatment.

## Data availability statement

The raw data supporting the conclusions of this article will be made available by the authors, without undue reservation.

## Author contributions

HH and XY designed this study and wrote the manuscript. RZ, XY, TZ, FW, JJ, and YX performed experiments. YK analyzed the data. All authors reviewed the results and approved the final version of the manuscript.

## Funding

This study was supported by research funding from the National Natural Science Foundation of China (82072328 and 81930112) and the Beijing Hospitals Authority Youth Programme (QML20211602).

## Conflict of interest

The authors declare that the research was conducted in the absence of any commercial or financial relationships that could be construed as a potential conflict of interest.

## Publisher's note

All claims expressed in this article are solely those of the authors and do not necessarily represent those of their affiliated organizations, or those of the publisher, the editors and the reviewers. Any product that may be evaluated in this article, or claim that may be made by its manufacturer, is not guaranteed or endorsed by the publisher.
